# Predicting responders to prone positioning in mechanically ventilated patients with COVID-19 using machine learning

**DOI:** 10.1186/s13613-022-01070-0

**Published:** 2022-10-20

**Authors:** Tariq A. Dam, Luca F. Roggeveen, Fuda van Diggelen, Lucas M. Fleuren, Ameet R. Jagesar, Martijn Otten, Heder J. de Vries, Diederik Gommers, Olaf L. Cremer, Rob J. Bosman, Sander Rigter, Evert-Jan Wils, Tim Frenzel, Dave A. Dongelmans, Remko de Jong, Marco A. A. Peters, Marlijn J. A. Kamps, Dharmanand Ramnarain, Ralph Nowitzky, Fleur G. C. A. Nooteboom, Wouter de Ruijter, Louise C. Urlings-Strop, Ellen G. M. Smit, D. Jannet Mehagnoul-Schipper, Tom Dormans, Cornelis P. C. de Jager, Stefaan H. A. Hendriks, Sefanja Achterberg, Evelien Oostdijk, Auke C. Reidinga, Barbara Festen-Spanjer, Gert B. Brunnekreef, Alexander D. Cornet, Walter van den Tempel, Age D. Boelens, Peter Koetsier, Judith Lens, Harald J. Faber, A. Karakus, Robert Entjes, Paul de Jong, Thijs C. D. Rettig, Sesmu Arbous, Sebastiaan J. J. Vonk, Tomas Machado, Willem E. Herter, Harm-Jan de Grooth, Patrick J. Thoral, Armand R. J. Girbes, Mark Hoogendoorn, Paul W. G. Elbers, The Dutch ICU Data Sharing Against COVID-19 Collaborators, Julia Koeter, Roger van Rietschote, M. C. Reuland, Laura van Manen, Leon Montenij, Jasper van Bommel, Roy van den Berg, Ellen van Geest, Anisa Hana, B. van den Bogaard, Peter Pickkers, Pim van der Heiden, Claudia(C. W.) van Gemeren, Arend Jan Meinders, Martha de Bruin, Emma Rademaker, Frits H. M. van Osch, Martijn de Kruif, Nicolas Schroten, Klaas Sierk Arnold, J. W. Fijen, Jacomar J. M. van Koesveld, Koen S. Simons, Joost Labout, Bart van de Gaauw, Michael Kuiper, Albertus Beishuizen, Dennis Geutjes, Johan Lutisan, Bart P. Grady, Remko van den Akker, Tom A. Rijpstra, Wim Boersma, Daniël Pretorius, Menno Beukema, Bram Simons, A. A. Rijkeboer, Marcel Aries, Niels C. Gritters van den Oever, Martijn van Tellingen, Annemieke Dijkstra, Rutger van Raalte, Ali el Hassouni, David Romero Guzman, Sandjai Bhulai, Dagmar M. Ouweneel, Ronald Driessen, Jan Peppink, G. J. Zijlstra, A. J. van Tienhoven, Evelien van der Heiden, Jan Jaap Spijkstra, Hans van der Spoel, Angelique M. E. de Man, Thomas Klausch, Robbert C. A. Lalisang, Michele Tonutti, Daan P. de Bruin, Mattia Fornasa, Michael de Neree tot Babberich, Olivier Thijssens, Lot Wagemakers, Hilde G. A. van der Pol, Tom Hendriks, Julie Berend, Virginia Ceni Silva, Robert F. J. Kullberg, Taco Houwert, Hidde Hovenkamp, Roberto Noorduijn Londono, Davide Quintarelli, Martijn G. Scholtemeijer, Aletta A. de Beer, Giovanni Cinà, Adam Izdebski, Leo Heunks, Nicole Juffermans, Arjen J. C. Slooter, Martijn Beudel

**Affiliations:** 1 Department of Intensive Care Medicine, Laboratory for Critical Care Computational Intelligence, Amsterdam Medical Data Science, Amsterdam UMC, Vrije Universiteit, Amsterdam, The Netherlands; 2 Quantitative Data Analytics Group, Department of Computer Science, Faculty of Science, VU University, Amsterdam, The Netherlands; 3 Department of Intensive Care, Erasmus Medical Center, Rotterdam, The Netherlands; 4 Intensive Care, UMC Utrecht, Utrecht, The Netherlands; 5 ICU, OLVG, Amsterdam, The Netherlands; 6 Department of Anesthesiology and Intensive Care, St. Antonius Hospital, Nieuwegein, The Netherlands; 7 Department of Intensive Care, Franciscus Gasthuis & Vlietland, Rotterdam, The Netherlands; 8 Department of Intensive Care Medicine, Radboud University Medical Center, Nijmegen, The Netherlands; 9 Department of Intensive Care Medicine, Amsterdam UMC, Amsterdam, The Netherlands; 10 Intensive Care, Bovenij Ziekenhuis, Amsterdam, The Netherlands; 11 Intensive Care, Canisius Wilhelmina Ziekenhuis, Nijmegen, The Netherlands; 12 Intensive Care, Catharina Ziekenhuis Eindhoven, Eindhoven, The Netherlands; 13 Department of Intensive Care, ETZ Tilburg, Tilburg, The Netherlands; 14 Intensive Care, HagaZiekenhuis, Den Haag, The Netherlands; 15 Intensive Care, Laurentius Ziekenhuis, Roermond, The Netherlands; 16 Department of Intensive Care Medicine, Northwest Clinics, Alkmaar, The Netherlands; 17 Intensive Care, Reinier de Graaf Gasthuis, Delft, The Netherlands; 18 Intensive Care, Spaarne Gasthuis, Haarlem en Hoofddorp, The Netherlands; 19 Intensive Care, VieCuri Medisch Centrum, Venlo, The Netherlands; 20 Intensive Care, Zuyderland MC, Heerlen, The Netherlands; 21 Department of Intensive Care, Jeroen Bosch Ziekenhuis, Den Bosch, The Netherlands; 22 Intensive Care, Albert Schweitzerziekenhuis, Dordrecht, The Netherlands; 23 ICU, Haaglanden Medisch Centrum, Den Haag, The Netherlands; 24 ICU, Maasstad Ziekenhuis Rotterdam, Rotterdam, The Netherlands; 25 ICU, SEH, BWC, Martiniziekenhuis, Groningen, The Netherlands; 26 Intensive Care, Ziekenhuis Gelderse Vallei, Ede, The Netherlands; 27 Department of Intensive Care, Ziekenhuisgroep Twente, Almelo, The Netherlands; 28 Department of Intensive Care, Medisch Spectrum Twente, Enschede, The Netherlands; 29 Department of Intensive Care, Ikazia Ziekenhuis Rotterdam, Rotterdam, The Netherlands; 30 Antonius Ziekenhuis Sneek, Sneek, The Netherlands; 31 Intensive Care, Medisch Centrum Leeuwarden, Leeuwarden, The Netherlands; 32 ICU, IJsselland Ziekenhuis, Capelle aan den IJssel, The Netherlands; 33 ICU, WZA, Assen, The Netherlands; 34 Department of Intensive Care, Diakonessenhuis Hospital, Utrecht, The Netherlands; 35 Department of Intensive Care, Adrz, Goes, The Netherlands; 36 Department of Anesthesia and Intensive Care, Slingeland Ziekenhuis, Doetinchem, The Netherlands; 37 Department of Anesthesiology, Intensive Care and Pain Medicine, Amphia Ziekenhuis, Breda, The Netherlands; 38 LUMC, Leiden, The Netherlands; 39 Pacmed, Amsterdam, The Netherlands

**Keywords:** COVID-19, Mechanical ventilation, Acute respiratory distress syndrome

## Abstract

**Background:**

For mechanically ventilated critically ill COVID-19 patients, prone positioning has quickly become an important treatment strategy, however, prone positioning is labor intensive and comes with potential adverse effects. Therefore, identifying which critically ill intubated COVID-19 patients will benefit may help allocate labor resources.

**Methods:**

From the multi-center Dutch Data Warehouse of COVID-19 ICU patients from 25 hospitals, we selected all 3619 episodes of prone positioning in 1142 invasively mechanically ventilated patients. We excluded episodes longer than 24 h. Berlin ARDS criteria were not formally documented. We used supervised machine learning algorithms Logistic Regression, Random Forest, Naive Bayes, K-Nearest Neighbors, Support Vector Machine and Extreme Gradient Boosting on readily available and clinically relevant features to predict success of prone positioning after 4 h (window of 1 to 7 h) based on various possible outcomes. These outcomes were defined as improvements of at least 10% in PaO_2_/FiO_2_ ratio, ventilatory ratio, respiratory system compliance, or mechanical power. Separate models were created for each of these outcomes. Re-supination within 4 h after pronation was labeled as failure. We also developed models using a 20 mmHg improvement cut-off for PaO_2_/FiO_2_ ratio and using a combined outcome parameter. For all models, we evaluated feature importance expressed as contribution to predictive performance based on their relative ranking.

**Results:**

The median duration of prone episodes was 17 h (11–20, median and IQR, *N* = 2632). Despite extensive modeling using a plethora of machine learning techniques and a large number of potentially clinically relevant features, discrimination between responders and non-responders remained poor with an area under the receiver operator characteristic curve of 0.62 for PaO_2_/FiO_2_ ratio using Logistic Regression, Random Forest and XGBoost. Feature importance was inconsistent between models for different outcomes. Notably, not even being a previous responder to prone positioning, or PEEP-levels before prone positioning, provided any meaningful contribution to predicting a successful next proning episode.

**Conclusions:**

In mechanically ventilated COVID-19 patients, predicting the success of prone positioning using clinically relevant and readily available parameters from electronic health records is currently not feasible. Given the current evidence base, a liberal approach to proning in all patients with severe COVID-19 ARDS is therefore justified and in particular regardless of previous results of proning.

**Supplementary Information:**

The online version contains supplementary material available at 10.1186/s13613-022-01070-0.

## Introduction

At the start of the coronavirus disease 2019 (COVID-19) pandemic, prone positioning quickly became an important treatment strategy in the armamentarium of intensivists [1]. This was based on physiological plausibility inferred from clinical experience and clinical trials of proning in non-COVID-19 ARDS [2]. Proning has been shown to reduce mortality in moderate-to-severe ARDS. Proposed physiological mechanisms include improved gas exchange and changed lung mechanics facilitating lung-protective ventilation [3]. Gravitational forces may lead to improved drainage of respiratory secretions, re-expansion of collapsed lung parenchyma, redistribution of aeration and pulmonary blood flow. This may improve lung compliance and improve ventilation–perfusion matching by reducing both shunting and dead space ventilation [3]. These effects in turn may facilitate lung-protective ventilation by reducing ventilator mechanical power while maintaining adequate gas exchange and therefore reduce the risk of ventilator induced lung injury [4].

However, proning is not without risks. Recognized adverse events include endotracheal tube obstruction and dislodgement, decreased clearance of mucus, and loss of venous access [5]. In addition, turning patients requires a coordinated team effort, which is a logistic challenge especially when operating at surge capacity in full personal protective equipment.

Therefore, predicting which critically ill COVID-19 patients will benefit from prone positioning may be of clinical value and it should come as no surprise that labeling of responders and non-responders quickly became common practice [1]. Response to proning is defined based on intermediate physiological measurements related to shunting, dead space ventilation and respiratory system compliance. As recently reviewed, this short-term physiological response is, on average, consistent with that known from non-COVID-19 ARDS [3]. And in contrast to non-COVID-19 ARDS the outcome in terms of survival was shown to be significantly better in responders than in non-responders [3].

We hypothesized that machine learning techniques known for their classifying power and predictive performance could be used on highly granular electronic health record data from critically ill COVID-19 patients to discriminate responders from non-responders. To perform these analyses, we focused on PaO_2_/FiO_2_ ratio, ventilatory ratio, respiratory system compliance and mechanical power as outcomes defining responsiveness. We used the Dutch Data Warehouse (DDW) which contains more than 3,000 critically ill COVID-19 patients in 25 hospitals in the Netherlands [6].

## Methods

The Medical Ethics Committee at Amsterdam UMC waived the need for patient informed consent and approved of an opt-out procedure for the collection of COVID-19 patient data during the COVID-19 crisis as documented under number 2020.156. This report adheres to the STROBE reporting guidelines [7].

### Patients

We selected all intubated ICU patients admitted during the first and second COVID-19 wave between March 2020 and February 2021 with at least one registration in prone position. Subsequent turns into a prone position were included and patients intubated for less than 24 h were excluded. Prone positioning events were excluded if prone duration was measured as longer than 24 h which could indicate inaccurate registration of turning events. Berlin ARDS criteria were not formally documented [8].

### Data preprocessing

Data from the DDW were filtered for unrealistic values (Additional file 1: Table S1). Individual measurements were aggregated for each hour starting from the admission time stamp. Parameters were forward filled for a variable amount of time based on clinical expertise following discussions with senior intensivists. For frequently measured parameters which are likely to be influenced by patient position, forward filling was limited to the time of position change (Additional file 1: Table S2). Missing values were derived (Additional file 1: Table S3). Further details may be found in Additional file 1.

### Outcome parameters

We created 5 different definitions to determine treatment success or failure for proning. We based these outcome definitions on PaO_2_/FiO_2_ ratio, ventilatory ratio, respiratory system compliance, and mechanical power, as well as a composite outcome. The composite outcome was defined as any improvement of 10% or more in PaO_2_/FiO_2_ ratio, ventilatory ratio or respiratory system compliance, without any deterioration of 10% or more in any of these parameters.

Target values were determined closest to 4 h after turning to a prone position. As clinical practice introduces some variance in the timing of measurements, measurements between 1 up to 7 h after proning were included. This time window was chosen as improvement was generally observable within a clinical shift and supported by the median difference in PaO_2_/FiO_2_ ratio for each hour after prone positioning (Additional file 1: Fig. S1).

Ventilatory ratio was calculated as (minute volume * PaCO_2_)/(predicted body weight * 100 * 37.5). [9] Mechanical power was calculated based on peak pressure and plateau pressure where available (Tidal Volume * (Peak Pressure – (0.5 * (Plateau Pressure – PEEP)) * Respiratory Rate * 0.1) or based on PEEP and pressure above PEEP otherwise (Tidal Volume * (PEEP + Pressure Above PEEP) * Respiratory Rate * 0.098) where pressure above PEEP is defined as peak inspiratory pressure minus PEEP [10–12] (Additional file 1: Table S3).

These target values were compared to values obtained in the 3 h prior to turning. Turning patients to a prone position was labeled successful based on an improvement in outcome parameters of 10% or greater. This cut-off value was chosen to allow for small improvements to still be regarded as favorable in the most critically ill patients, while requiring a more pronounced effect in patients with more favorable physiology. Re-supination within 4 h after pronation was labeled as failure. As the time of registration of patient positioning may deviate from the actual moment of change in position, measurements in the same hour as registration of position changes were discarded from analysis.

As a sensitivity analysis, we used 20% as a cut-off. We also used 20 mmHg as a cut-off for change on PaO_2_/FiO_2_ ratio in line with previous research [3]. In addition, we also created a second combined outcome defined as an increase of at least 10% in either PaO_2_/FiO_2_ ratio, ventilatory ratio or respiratory system compliance without a deterioration of more than 10% in the other two. This new combined outcome served to represent a bedside definition of proning success where proning should at least improve one physiological measure with a plausible association with outcome while not seriously worsen the clinical picture.

### Features

Based on a combination of clinical expertise and correlation with PaO_2_/FiO_2_ ratio difference, a set of 80 candidate features were selected to prevent overfitting on too many features. These include feature augmentations created as rolling 2-h averages and 8-h slopes, as well as the last values of each outcome shortly before turning to a prone position (Additional file 1: Table S4). Missing data were imputed using median imputation for continuous features and imputed as absent for medical history. To ensure the inclusion of clinically essential parameters, we combined the data for specific sub-parameters [9, 10]. Static and dynamic respiratory compliance were aggregated into a single compliance parameter where static compliance took precedence over dynamic compliance if both were available. Furthermore, driving pressure and pressure above PEEP were combined into a delta-pressure parameter. Previous medical history was aggregated into two groups based on respiratory system involvement as these were deemed likely to have a comparable effect on the outcome parameters. Finally, if a previous proning event was labeled as successful, the patient was marked as a previous responder to prone positioning in the next event. Numeric data were normalized for each analysis. Further details may be found in the online Additional file 1.

### Modeling

Data were split in a training and test set, where subsequent turns of the same patient were kept in the same set to prevent leakage of information. For classification modeling, we used logistic regression, Random Forest, K-Nearest Neighbors (KNN), Support Vector Machines (SVM), Gaussian Naive Bayes (GNB) and XGBoost (XGB) for each of the classification targets. These models were selected based on their general predictive performance and prevalence in medical literature [13–15]. For each of the models, hyper-parameter optimization was performed using a grid search on the training set. Contribution to the predictions was evaluated using feature importances, permutation importances and absolute coefficients (Additional file 1: Tables S5, S6).

## Results

1142 out of a total of 3600 patients were recorded with at least one prone position, with a total of 3619 prone events with a median of 2 (IQR 1–4) prone events per patient with a maximum of 27 prone events. The median last PaO_2_/FiO_2_ ratio in the last 3 h before turning to a prone position was 112 mmHg (IQR 87–142, *N* = 2958). At 4 h post-prone positioning, the median difference in PaO_2_/FiO_2_ ratio was 14.9 (IQR − 5–41, *N* = 2211). Time spent in a prone position was 17 h (11–20, median and IQR, *N* = 2632) (Table 1). PEEP levels before prone positioning were 12 (10–14.3, median and IQR, *N* = 3332) and after prone positioning were 12 (10–14.3, median and IQR, *N* = 3303). The overall ICU mortality of patients having spent time in a prone position was 424 out of 1142 (37.1%) with a median length of stay of 15.6 days (9–26.5, median and IQR). Further details can be found in Additional file 1 (Additional file 1: Table S7).

**Table 1:** Patient characteristics including clinically relevant features used for prediction

Parameter	Missing (%)	Values	Units
Number of prone events		3619	
Number of patients		1142	
Prone events per patient, median [Q1, Q3]		2 [1, 4]	
Age, median [Q1,Q3]	0.0	66.0 [58.0,72.0]	Years
Gender, female, n (%)	0.0	944 (26.1)	
BMI, median [Q1,Q3]	0.0	27.2 [24.6,30.0]	
*Comorbidities*			
Chronic dialysis, n (%)	30.0	43 (1.7)	
Chronic renal insufficiency, n (%)	30.0	256 (10.1)	
Cirrhosis, n (%)	30.0	103 (4.1)	
COPD, n (%)	30.0	349 (13.8)	
Diabetes, n (%)	30.0	613 (24.2)	
Neoplasm, n (%)	30.0	132 (5.2)	
Hematologic malignancy, n (%)	30.0	193 (7.6)	
Immune insufficiency, n (%)	30.0	356 (14.1)	
Respiratory insufficiency, n (%)	30.0	196 (7.7)	
Cardiovascular insufficiency, n (%)	30.0	131 (5.2)	
*Clinical measurements, average*			
etCO_2_ 2 h, mean (SD)	14.6	41.3 (11.0)	mmHg
paCO_2_-etCO_2_ 2 h, mean (SD)	27.5	13.3 (10.9)	mmHg
FiO_2_ 2 h, mean (SD)	3.3	65.6 (17.6)	%
Peak airway pressure 2 h, mean (SD)	10.3	27.2 (6.2)	cmH_2_O
Delta pressure 2 h, mean (SD)	20.3	13.5 (5.4)	cmH_2_O
PEEP 2 h, mean (SD)	8.1	12.2 (3.3)	cmH_2_O
Respiratory system compliance 2 h, mean (SD)	10.2	43.6 (31.0)	ml/cmH_2_O
Tidal volume per kg 2 h, mean (SD)	8.2	6.7 (1.5)	ml/kg
Respiratory rate 2 h, mean (SD)	1.5	24.7 (5.5)	/min
Minute volume 2 h, mean (SD)	8.3	11.0 (3.0)	ml/min
PaO_2_/FiO_2_ ratio 2 h, mean (SD)	20.4	120.5 (45.4)	mmHg
Ventilatory ratio 2 h, mean (SD)	12.6	2.3 (0.9)	
Mechanical power 2 h, mean (SD)	12.5	32.9 (14.3)	J/min
Mean arterial blood pressure 2 h, mean (SD)	3.2	78.7 (12.5)	mmHg
Heart rate 2 h, mean (SD)	5.9	88.5 (19.6)	/min
CRP 2 h, mean (SD)	21.0	199.2 (123.8)	mg/l
Leukocytes 2 h, mean (SD)	6.5	11.5 (5.5)	10^9^/l
Thrombocytes 2 h, mean (SD)	4.8	304.9 (135.1)	10^9^/l
d-dimer 2 h, mean (SD)	57.7	3061.2 (2251.5)	ng/ml
Lactate arterial 2 h, mean (SD)	44.0	1.3 (0.6)	mmol/l
pO_2_ arterial 2 h, mean (SD)	19.5	69.7 (10.2)	mmHg
pCO_2_ arterial 2 h, mean (SD)	6.8	53.9 (14.3)	mmHg
pH arterial 2 h, mean (SD)	24.8	7.4 (0.1)	
Creatinine 2 h, mean (SD)	6.0	115.8 (105.2)	µmol/l
*Clinical measurements, slopes*			
Last FiO_2_ 2 h slope, median [Q1,Q3]	13.5	0.0 [− 5.0,2.5]	%/h
PaO_2_/FiO_2_ ratio 8 h slope, median [Q1,Q3]	80.2	1.0 [− 1.7,3.7]	mmHg/h
Ventilatory ratio 8 h slope, median [Q1,Q3]	47.6	− 0.0 [− 0.0,0.0]	/h
Mechanical power 8 h slope, median [Q1,Q3]	32.0	0.0 [− 0.6,0.6]	J/min/h
*Outcomes*			
PaO_2_/FiO_2_ ratio 3 h last, median [Q1,Q3]	18.3	111.6 [86.8,141.7]	
Ventilatory ratio 3 h last, median [Q1,Q3]	32.2	2.2 [1.7,2.9]	
Mechanical power 3 h last, median [Q1,Q3]	23.5	32.0 [20.9,44.7]	J/min
Respiratory system compliance 3 h last, median [q1, q3]	9.8	32.8 [24.4,48.5]	ml/cmH_2_O
PaO_2_/FiO_2_ ratio difference, median [Q1, Q3]	27.3	14.9 [− 5.3,40.5]	
Ventilatory ratio difference, median [Q1, Q3]	37.4	0.0 [− 0.3,0.4]	
Mechanical power difference, median [Q1, Q3]	26.7	0.2 [− 9.8,9.2]	J/min
Respiratory system compliance difference, median [Q1, Q3]	12.9	− 0.6 [− 5.8,3.6]	ml/cmH_2_O

For clinical parameters, the mean of the past 2 h was aggregated per patient and slopes were calculated based on mean values in the hour before proning

This initial dataset of prone events was reduced for each outcome to contain only observations with a measured outcome. This resulted in a dataset of 1289 prone events for the composite outcome, 1820 prone events for PaO_2_/FiO_2_ ratio outcome, 1626 prone events for the ventilatory ratio outcome, 1829 prone events for the mechanical power outcome and 2140 prone events for the compliance outcome. Outcome labels were balanced for predicting PaO_2_/FiO_2_ ratios (52.3% success rate) up to moderately imbalanced for predicting the composite outcome (15.8% success rate).

**Fig. 1:**
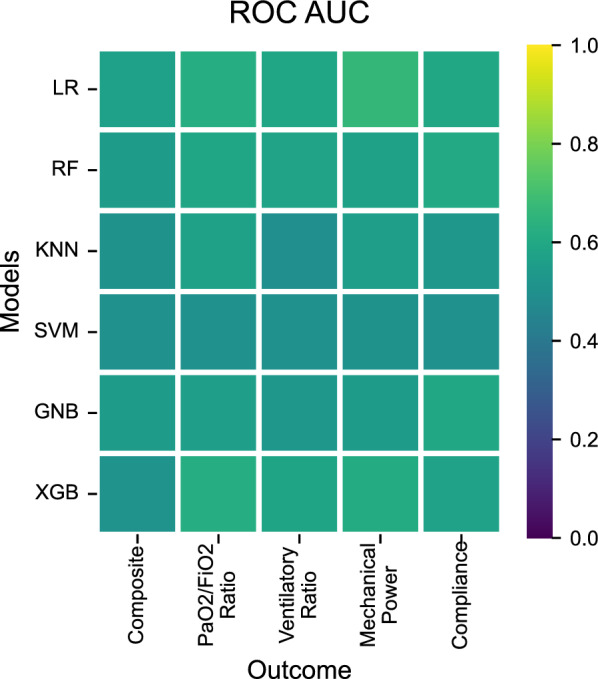
Model performance by ROC AUC score for predicting improvement in various outcome parameters after turning patients to a prone position. The ROC AUC compares the true positive rate to the false positive rate where a performance of 1.0 reflects perfect scores where 0.5 describes complete randomness. *LR* logistic regression, *RF*  random forest, *KNN*  K-Nearest Neighbors, *SVM*  support vector machine, *GNB* Gaussian Naïve Bayes, *XGB*  eXtreme Gradient Boosting

**Fig. 2:**
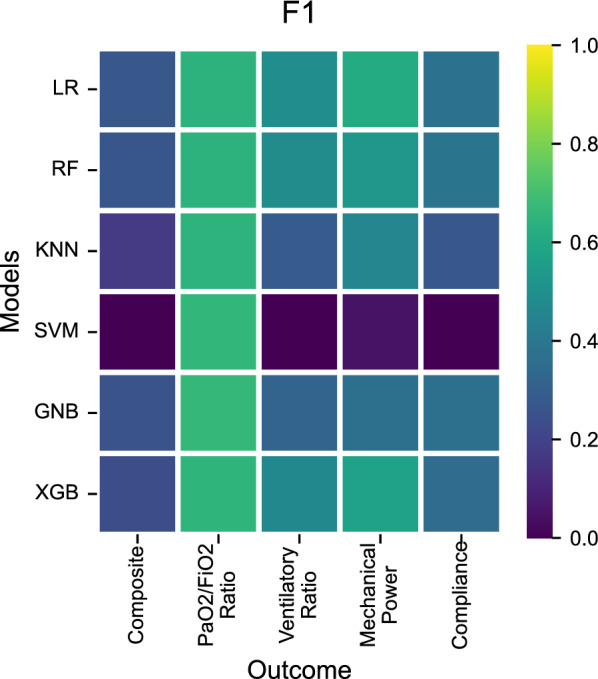
Model performance by F1-score for predicting improvement in various outcome parameters after turning patients to a prone position. The F1-score combines the precision (positive predictive value) and recall (sensitivity) scores to provide a single metric to compare model performance where a performance of 1.0 reflects perfect scores while 0.0 reflects the worst performance. *LR* logistic regression, *RF*  random forest, *KNN*  K-Nearest Neighbors, *SVM*  support vector machine, *GNB* Gaussian Naïve Bayes, *XGB*  eXtreme Gradient Boosting

Predictive performance varied across models and outcomes with the most accurate predictions originating from Logistic Regression, Random Forest and XGBoost on relative improvement of PaO_2_/FiO_2_ ratio, based on a comparable 0.59–0.62 area under the receiver operator characteristic curve (ROC AUC), while the Gaussian Naive Bayes and Support Vector Machine provided an ROC AUC close to 0.5 (Fig. 1, Additional file 1: Table S8). For other outcomes, the ROC AUC was relatively close to 0.5. The F1-score, interpreted as the weighted average of the precision and recall values, was between 0.64 and 0.67 for all models predicting PaO_2_/FiO_2_ ratio, but lower for most models on the other outcomes with the exception of logistic regression (0.61) and XGBoost (0.58) for mechanical power (Fig. 2, Additional file 1: Table S9). Results were similar for sensitivity analyses where the cut-off was set to 20% or 20 mmHg, and for sensitivity analyses where no minimal prone duration was required (Additional file 1: Tables S10, S11).

Underlying contribution of features to the predictive performance was generally low and showed little consistency across models for the most predictive features. (Table 2, Additional file 1: Tables S12, S13). Correlation between successful outcome of a previous proning episode and successful outcome of a subsequent proning episode was virtually absent. Correlation between PEEP levels before turning to a prone position and a successful outcome was absent as well (Table 3).

**Table 2:** Features ranked by feature importance, permutation importance or absolute coefficient values for outcome PaO_2_/FiO_2_ prediction

	*LR*	*RF*	*GNB*	*XGB*	*Mean rank*
PaO_2_/FiO_2_ ratio 3 h last	73	73	73	73	73
last FiO_2_ 2 h slope	72	70	64	70	69
PaO2/FiO_2_ ratio 2 h	65	72	70	44	62.75
Ventilatory ratio 8 h slope	69	49	61.5	66	61.375
PaO_2_ 2 h	61	71	72	36	60

The top parameters scored consistently high across the various models which may possibly indicate some predictive capacity for these features

**Table 3:** Correlation of previous response to prone positioning or PEEP levels with the outcome label for each of the outcomes

Outcome	Correlation responder	Correlation PEEP
Composite, relative 10%	− 0.020	− 0.017
Composite, absolute	− 0.027	− 0.007
PaO_2_/FiO_2_ ratio, relative 10%	0.063	0.027
PaO_2_/FiO_2_, absolute	0.152	0.027
Ventilatory ratio, relative 10%	− 0.013	− 0.007
Mechanical power, relative 10%	0.068	0.009
Compliance, relative 10%	0.117	− 0.026

Relative indicates a relative improvement of 10% in PaO_2_/FiO_2_ ratio, while absolute indicates an improvement of 20 mmHg

## Discussion

This is the first study to attempt to use machine learning techniques in predicting prone positioning responsiveness in intubated critically ill patients with COVID-19 using routinely registered data in the electronic health records. The authors are also not aware of any papers using traditional statistical regression-based techniques to infer predictors for the success of prone positioning. Despite extensive modeling using a plethora of machine learning techniques and inclusion of a large number of potentially clinically relevant features, discrimination between responders and non-responders based on commonly used physiological outcomes remained poor.

Notably, not even being a previous responder to prone positioning showed any meaningful contribution to the prediction of a next response. While expecting a similar response as before may seem intuitive, lung and respiratory system physiology can change rapidly with progression of disease or through the effect of therapy. Therefore, relying on previous success or failure may be suboptimal.

These findings of poor predictive performance in the context of suggested, although debated, benefit from prone positioning including mortality, based on previous literature, are important because of their clinical implication. In this context, prone positioning should not be withheld in mechanically ventilated COVID-19 patients based on their characteristics or previous proning failure despite the extra work involved and potential adverse effects [3].

Among the most consistently important features were the last known PaO_2_/FiO_2_ ratio, along with FiO_2_ slopes. However, their impact varied greatly among models and predictive performance remained poor (Additional file 1: Tables S12, S13).

Mortality in proned COVID-19 patients was 424 out of 1142 patients (37.1%) in this study, while overall mortality for all mechanically ventilated COVID-19 patients was previously shown to be circa 30%, and overall ICU mortality for all COVID-19 patients was 24.4% [6, 16]. This trend is expected as each step corresponds to an increased severity of disease and thus a decreased chance of survival.

In one study on COVID-19 ARDS, survival was better in responders than in non-responders to proning in terms of oxygenation [3]. However, this relationship is subject to debate for non-COVID-19 ARDS. Notably, in the landmark PROSEVA trial that showed an important mortality benefit for proning, there was no evidence that this mortality effect only accrues in patients who show a physiological response to proning [2, 17]. In our study, the association between response in P/F ratio and survival was weak. This should encourage a liberal approach to proning regardless of short-term improvements in physiology given the evidence supporting its effect on survival.

Strengths of this study include the use of large amounts of routinely collected granular data from a large multi-center database, the rigorous and extensive modeling attempts and the broad approach to defining short-term response to proning based on the principles of shunting, dead space ventilation and respiratory system compliance.

However, the physiological effect of proning may be difficult to predict at least partly due to limitations inherent in the chosen outcome variables. Ventilatory ratio is a poor surrogate for dead space ventilation and ventilation/perfusion match. PaO_2_/FiO_2_ ratio is a relatively poor surrogate for shunt and ventilation/perfusion match. Pressure above PEEP is a weak surrogate for plateau pressure, feeding into static respiratory system compliance, which is itself a poor surrogate for lung compliance.

Defining the exact cut-off for success is therefore non-trivial. Based on previous literature, we may select an absolute change of at least 20 mmHg [3]. But a relative change may be closer to clinical practice in which physicians may still consider smaller improvement successful in the most severe cases. Nevertheless, sensitivity analyses showed no major differences in predictive performance when adjusting these cut-off values.

This study also comes with limitations. For some potential features, data availability was limited due to varying frequencies of measurements and imputation strategies were necessary. Also imaging data as well as measurements requiring maneuvers such as inspiratory hold were mostly unavailable. Individual data points were not manually validated due to the vast amount of data, although most evident data entry errors were removed through preprocessing. These models were trained on 25 different hospitals, but external validation is needed to generalize these findings. Furthermore, as disease and treatment change over time, this drifting data may influence future applicability. Finally, our absence of evidence should not be regarded as evidence of absence. It is certainly thinkable that future developments in machine learning combined with increasing availability of data might facilitate better discrimination between physiological responders and non-responders.

## Conclusion

The physiological response to prone positioning of COVID-19 ARDS patients could not be reliably predicted with highly granular EHR data using novel machine learning techniques. Predictors for physiological improvement were inconsistent and earlier response to proning showed no correlation to future responses. Although a definitive proof of unpredictability cannot be provided, we have shown that current EHR data are insufficient to aid in the decision to turn patients to a prone position. Therefore, the decision to turn a patient to a prone position should be based on group level evidence and only be omitted based on individual contra-indications.

## Supplementary Information

**Additional file 1: Table S1.** Parameter bounds. **Table S2.** Parameter categories. **Table S3.** Feature derivations. **Table S4.** Included features. **Table S5.** Hyper parameter optimization. **Table S6.** Feature Importance Metrics. **Table S7.** Patient characteristics. **Table S8.** ROC AUC score. **Table S9.** F1-score. **Table S10.** Sensitivity Analysis Minimal Prone Duration. **Table S11.** Sensitivity Analysis Cut-off Values. **Table S12.** Ranked Feature Importance. **Table S13.** Feature Importance Values. **Figure S1.** PaO_2_/FiO_2_ ratio difference in mmHg over time after starting prone position. Prone positions which continued for longer than 24 hours were filtered out.

## Data Availability

The dataset supporting the conclusions of this article is available in the Dutch Data Warehouse, 10. 1186/s13054-021-03733-z https://amsterdammedicaldatascience.nl [6, 18]. Source code for this manuscript is available upon request.

## Abbreviations

ARDS: Acute respiratory distress syndrome
COVID-19: Coronavirus disease 2019
DDW: Dutch Data Warehouse
GNB: Gaussian Naive Bayes
IQR: Interquartile range
KNN: K-nearest neighbors
Q1: First quartile
Q3: Third quartile
ROC AUC: Area under the receiver-operator characteristic curve
SD: Standard deviation
SVM: Support vector machine
XGB: Extreme gradient boosting
