# Supplementary material for: Predicting responders to prone positioning in mechanically ventilated patients with COVID-19 using machine learning
Source: Ann Intensive Care. 2022 Oct 20;12:99. doi: 10.1186/s13613-022-01070-0 (PMC9583049; doi:10.1186/s13613-022-01070-0)
Supplement: Supplementary file 1 — Additional file 1: Table S1. Parameter bounds. Table S2. Parameter categories. Table S3. Feature derivations. Table S4. Included features. Table S5. Hyper parameter optimization. Table S6. Feature Importance Metrics. Table S7. Patient characteristics. Table S8. ROC AUC score. Table S9. F1-score. Table S10. Sensitivity Analysis Minimal Prone Duration. Table S11. Sensitivity Analysis Cut-off Values. Table S12. Ranked Feature Importance. Table S13. Feature Importance Values. Figure S1. PaO2/FiO2 ratio difference in mmHg over time after starting prone position. Prone positions which continued for longer than 24 hours were filtered out. [file 13613_2022_1070_MOESM1_ESM.docx]

Online Supplementary Material

Predicting PaO2/FiO2 ratio improvement in prone position: a machine learning approach

The Dutch ICU Data Sharing Collaborators

# Tables

## Supplementary Table 1. Parameter bounds

Data was filtered based on upper and lower bounds and values outside of these bounds were dropped. For specific parameters, the dynamic filters were used as these values would have to correspond with each other. For example, if plateau pressure was lower than PEEP, then plateau the value for plateau pressure was removed.

| **Parameter** | **Lower bound** | **Upper bound** |
| --- | --- | --- |
| ***Static filters*** |  |  |
| FiO2 | 0.21 | 1.00 |
| PaO2/FiO2 ratio | 0 | 480 |
| PaO2 | 0 | 100 |
| PaCO2 | 0 | 100 |
| etCO2 | 0 | 100 |
| Tidal Volume | 50 | 1000 |
| Minute Volume | 1 | 25 |
| Compliance | 0 | 200 |
| Plateau Pressure | 0 | 50 |
| Driving Pressure | 0 | 50 |
| PEEP | 0 | 50 |
| Physiologic dead space | 0 | 3000 |
| Central venous pressure | 0 | 50 |
| Base excess | -50 | 50 |
|  |  |  |
| ***Dynamic filters*** | **Formula** |  |
| Plateau Pressure | Plateau Pressure > PEEP |  |
| Driving Pressure | Driving Pressure = Plateau Pressure – PEEP |  |
| PaCO2 – etCO2 | Pa-etCO2 = PaCO2-etCO2 |  |

## Supplementary Table 2. Parameter categories

Parameters were grouped in clinical categories. For each category, a limit was specified for the duration these parameters could be forward filled to impute missing data. If respect positioning was True, then forward filling was only allowed within the same prone or supine position as these variables were likely to change depending on the position of the patient.

| **Categories** | **Parameters** | **Forward Fill Duration** | **Respect positioning** |
| --- | --- | --- | --- |
| Patient Characteristics | Height, Weight, BMI | unlimited | False |
| Respiratory | All | 3 hours | True |
| Circulatory | All | 4 hours | True |
| Laboratory | CRP, Leukocytes, Thrombocytes, Creatinine, Sodium, Chloride | 26 hours | False |
| Laboratory | Remaining laboratory parameters | 26 hours | True |

## Supplementary Table 3. Feature derivations

Parameters which could be calculated based on their component were derived according the specified formulas. For parameters in which components can be exchanged, we derived all components by transforming the specified formulas.

| **Feature** | **Source parameters** | **Formula** | **Method** |
| --- | --- | --- | --- |
| Driving Pressure | Plateau Pressure PEEP | Plateau Pressure - PEEP | Derive all components |
| etCO2 – PaCO2 gap | PaCO2, etCO2 | etCO2 – PaCO2 | Derive all components |
| Tidal Volume | Minute Volume, Respiratory Rate | Minute Volume / Respiratory Rate | Derive all components |
| Static Compliance | Tidal Volume, Driving Pressure | Tidal Volume / Driving Pressure | Derive all components |
| Dynamic Compliance | Tidal Volume, Peak Pressure, PEEP | Tidal Volume / (Peak Pressure – PEEP) | Derive all components |
| BMI | Height, Weight, Tidal Volume | Weight / (height^2) | Derive all components |
| Predicted Body Weight | Sex, height, Tidal Volume, Tidal Volume per kg PBW | 1. (50 + (-4.5 * sex)) + (0.91 * ((height * 100) - 152.4)) 2. Tidal Volume / Tidal Volume per kg PBW | Derive all components |
| PaO2/FiO2 ratio | FiO2, PaO2 | PaO2 / FiO2 | Derive all components |
| Ventilatory Ratio | Minute Volume, PaCO2, Predicted Body Weight | (Minute Volume * PaCO2) / (Predicted Body Weight * 100 * 37.5) [1] | Derive all components |
| Mechanical Power, in Volume Controlled Ventilation | Tidal Volume, Peak Pressure, Plateau Pressure, PEEP, Respiratory Rate | Tidal Volume * (Peak Pressure – (0.5 * (Plateau Pressure – PEEP)) * Respiratory Rate * 0.1 [2] | Derive one way only |
| Mechanical Power, in Pressure Controlled Ventilation | Tidal Volume, PEEP, Pressure Above PEEP, Respiratory Rate | Tidal Volume * (PEEP + Pressure Above PEEP) * Respiratory Rate * 0.098 [2] | Derive one way only |
| Delta-Pressure | Driving Pressure, Pressure Above PEEP | Driving Pressure > Pressure Above PEEP | Combine values if missing |
| Compliance | Static Compliance, Dynamic Compliance | Static Compliance > Dynamic Compliance | Combine values if missing |

## Supplementary Table 4. Included features

Table of included features with their respective groups corresponding to the data processing groups of Supplementary Table 2 and the resulting aggregations which were used in predictive modeling.

| **Group** | **Parameter** | **Type** |
| --- | --- | --- |
| **Static** | Age, Gender, BMI, Previous medical history categories |  |
| **Respiratory** | PaO2/FiO2 ratio | 2h mean, 8h slope, 3h last |
|  | FiO2 | 2h mean, 8h slope, 2h last |
|  | O2 Saturation | 2h mean, 8h slope |
|  | End tidal CO2 | 2h mean, 8h slope |
|  | PaCO2-etCO2 | 2h mean, 8h slope |
|  | Peak airway pressure | 2h mean, 8h slope |
|  | Delta Pressure (combined) | 2h mean, 8h slope |
|  | Mean airway pressure | 2h mean, 8h slope |
|  | PEEP | 2h mean, 8h slope |
|  | Mechanical Power | 2h mean, 8h slope, 3h last |
|  | Ventilatory Ratio | 2h mean, 8h slope, 3h last |
|  | Lung Compliance (combined) | 2h mean, 8h slope, 3h last |
|  | Tidal Volume / kg | 2h mean, 8h slope |
|  | Respiratory rate | 2h mean, 8h slope |
|  | Minute Volume | 2h mean, 8h slope |
|  | Responder | Boolean based on previous prone position |
| **Circulatory** | Blood Pressure, arterial | 2h mean, 8h slope |
|  | Heart rate | 2h mean, 8h slope |
|  | Temperature | 2h mean, 8h slope |
| **Laboratory** | pH, arterial | 2h mean, 8h slope |
|  | PaO2, arterial | 2h mean, 8h slope |
|  | PaCO2, arterial | 2h mean, 8h slope |
|  | Base Excess | 2h mean, 8h slope |
|  | Lactate, arterial | 2h mean, 8h slope |
|  | D-Dimer | 2h mean, 24h slope |
|  | CRP | 2h mean, 24h slope |
|  | Leukocytes | 2h mean, 24h slope |
|  | Thrombocytes | 2h mean, 24h slope |
|  | Creatinine | 2h mean, 24h slope |
|  | Sodium | 2h mean |
|  | Chloride | 2h mean |
|  | Albumin | 2h mean |
|  | Bilirubin total | 2h mean |

## Supplementary Table 5. Hyper parameter optimization

Hyper parameters were optimized for each model based on a grid search using model-specific hyper parameters and values. Optimizing these hyper parameters is needed to find a balance between improving accuracy and preventing overfitting. For each individual outcome analysis, this grid search was performed separately.

| **Type** | **Model** | **Grid** |
| --- | --- | --- |
| Classification | Logistic Regression | C: [0.1, 0.5, 1.0] |
| Classification | Random Forest | max_depth: [10, 30, 70, 100]  max_features: [‘auto’, ‘sqrt’]  min_samples_leaf: [1, 2, 4]  min_samples_split: [2, 5, 10]  n_estimators: [100, 200, 400, 800] |
| Classification | KNN | leaf_size: [5, 10, 50]  n_neighbours: [5, 10, 30]  p: [1, 2] |
| Classification | SVM | C: [0.1, 0.5, 1.0]  Kernel: [‘rbf’] |
| Classification | GNB | var_smoothing: [0.1, 0.01, 0.001] |
| Classification | XGBoost | Max_depth: [5, 10, 20, 50] |

## Supplementary Table 6. Feature Importance Metrics

Feature importance describes the contributing influence of each feature on the final prediction for each model. However, these values cannot be compared directly as the exact method for calculating feature importance varies for each model. The metric specifies the exact algorithm used for calculating the respective importance.

| **Model** | **Type** | **Metric** |
| --- | --- | --- |
| LR | Coefficients | - |
| RF | Feature Importance | Gini |
| GNB | Permutation Importance | F1-score |
| XGB | Feature Importance | Gain |

## Supplementary Table 7. Patient characteristics

Patient characteristics for the full dataset of prone patients. PEEP = Positive End-Expiratory Pressure.

| **Parameter** | **Missing (%)** | **Values** | **Units** |
| --- | --- | --- | --- |
| number of prone events |  | 3619 |  |
| Number of patients |  | 1142 |  |
| Prone events per patient, median [Q1, Q3] |  | 2 [1, 4] |  |
| age, median [Q1,Q3] | 0.0% | 66.0 [58.0,72.0] | years |
| gender, female, n (%) | 0.0% | 944 (26.1) |  |
| bmi, median [Q1,Q3] | 0.0% | 27.2 [24.6,30.0] | |
| chronic dialysis, mean (SD) | 30.0% | 0.0 (0.1) |  |
| chronic renal insufficiency, mean (SD) | 30.0% | 0.1 (0.3) |  |
| cirrhosis, mean (SD) | 30.0% | 0.0 (0.2) |  |
| COPD, mean (SD) | 30.0% | 0.1 (0.3) |  |
| diabetes, mean (SD) | 30.0% | 0.2 (0.4) |  |
| neoplasm, mean (SD) | 30.0% | 0.1 (0.2) |  |
| hematologic malignancy, mean (SD) | 30.0% | 0.1 (0.3) |  |
| immune insufficiency, mean (SD) | 30.0% | 0.1 (0.3) |  |
| respiratory insufficiency, mean (SD) | 30.0% | 0.1 (0.3) |  |
| cardiovascular insufficiency, mean (SD) | 30.0% | 0.1 (0.2) |  |
| etCO2 2h, mean (SD) | 14.6% | 41.3 (11.0) | mmHg |
| paCO2-etCO2 2h, mean (SD) | 27.5% | 13.3 (10.9) | mmHg |
| FiO2 2h, mean (SD) | 3.3% | 65.6 (17.6) | % |
| peak airway pressure 2h, mean (SD) | 10.3% | 27.2 (6.2) | cmH2O |
| mean airway pressure 2h, mean (SD) | 14.8% | 17.2 (4.2) | cmH2O |
| delta pressure 2h, mean (SD) | 9.0% | 13.7 (5.4) | cmH2O |
| PEEP 2h, mean (SD) | 8.1% | 12.2 (3.3) | cmH2O |
| lung compliance 2h, mean (SD) | 10.2% | 43.6 (31.0) | ml/cmH2O |
| tidal volume per kg 2h, mean (SD) | 8.2% | 6.7 (1.5) | ml/kg |
| respiratory rate 2h, mean (SD) | 1.5% | 24.7 (5.5) | /min |
| minute volume 2h, mean (SD) | 8.3% | 11.0 (3.0) | ml/min |
| PaO2/FiO2 ratio 2h, mean (SD) | 20.4% | 120.5 (45.4) | |
| ventilatory ratio 2h, mean (SD) | 12.6% | 2.3 (0.9) |  |
| mechanical power 2h, mean (SD) | 12.5% | 32.9 (14.3) | J/min |
| O2 saturation 2h, mean (SD) | 3.0% | 92.6 (3.1) | % |
| mean arterial blood pressure 2h, mean (SD) | 3.2% | 78.7 (12.5) | mmHg |
| heart rate 2h, mean (SD) | 5.9% | 88.5 (19.6) | /min |
| temperature 2h, mean (SD) | 12.5% | 37.3 (1.0) | °C |
| CRP 2h, mean (SD) | 21.0% | 199.2 (123.8) | mg/l |
| leukocytes 2h, mean (SD) | 6.5% | 11.5 (5.5) | 10^9/l |
| thrombocytes 2h, mean (SD) | 4.8% | 304.9 (135.1) | 10^9/l |
| d-dimer 2h, mean (SD) | 57.7% | 3061.2 (2251.5) | ng/ml |
| lactate arterial 2h, mean (SD) | 44.0% | 1.3 (0.6) | mmol/l |
| pO2 arterial 2h, mean (SD) | 19.5% | 69.7 (10.2) | mmHg |
| pCO2 arterial 2h, mean (SD) | 6.8% | 53.9 (14.3) | mmHg |
| pH arterial 2h, mean (SD) | 24.8% | 7.4 (0.1) |  |
| creatinine 2h, mean (SD) | 6.0% | 115.8 (105.2) | µmol/l |
| albumin 2h, mean (SD) | 39.2% | 22.1 (5.4) | g/l |
| base excess 2h, mean (SD) | 9.8% | 4.2 (5.9) | mmol/l |
| bilirubin 2h, mean (SD) | 30.5% | 12.1 (18.6) | µmol/l |
| chloride 2h, mean (SD) | 18.8% | 105.6 (5.7) | mmol/l |
| sodium 2h, mean (SD) | 4.4% | 142.6 (5.8) | mmol/l |
| etCO2 8h slope, median [Q1,Q3] | 31.4% | -0.1 [-0.7,0.4] | mmHg/h |
| paCO2-etCO2 8h slope, median [Q1,Q3] | 73.4% | -0.3 [-1.0,0.3] | mmHg/h |
| FiO2 8h slope, median [Q1,Q3] | 18.6% | -0.9 [-2.5,0.0] | %/h |
| last FiO2 2h slope, median [Q1,Q3] | 13.5% | 0.0 [-5.0,2.5] | %/h |
| peak airway pressure 8h slope, median [Q1,Q3] | 28.2% | -0.0 [-0.4,0.1] | cmH2O/h |
| mean airway pressure 8h slope, median [Q1,Q3] | 32.4% | -0.0 [-0.3,0.1] | cmH2O/h |
| delta pressure 8h slope, median [Q1,Q3] | 24.6% | 0.0 [-0.3,0.1] | cmH2O/h |
| PEEP 8h slope, median [Q1,Q3] | 24.0% | 0.0 [-0.1,0.0] | cmH2O/h |
| lung compliance 8h slope, median [Q1,Q3] | 29.7% | 0.2 [-0.4,1.0] | ml/cmH2O/h |
| tidal volume per kg 8h slope, median [Q1,Q3] | 27.4% | 0.0 [-0.1,0.1] | ml/kg/h |
| respiratory rate 8h slope, median [Q1,Q3] | 15.8% | 0.0 [-0.2,0.2] | /min/h |
| minute volume 8h slope, median [Q1,Q3] | 27.7% | 0.0 [-0.1,0.2] | ml/min/h |
| PaO2/FiO2 ratio 8h slope, median [Q1,Q3] | 80.2% | 1.0 [-1.7,3.7] | /h |
| ventilatory ratio 8h slope, median [Q1,Q3] | 47.6% | -0.0 [-0.0,0.0] | /h |
| mechanical power 8h slope, median [Q1,Q3] | 32.0% | 0.0 [-0.6,0.6] | J/min/h |
| O2 saturation 8h slope, median [Q1,Q3] | 19.8% | -0.1 [-0.4,0.2] | %/h |
| mean arterial blood pressure 8h slope, median [Q1,Q3] | 20.7% | -0.1 [-1.2,0.9] | mmHg/h |
| heart rate 8h slope, median [Q1,Q3] | 23.0% | -0.1 [-1.2,0.9] | /min/h |
| temperature 8h slope, median [Q1,Q3] | 46.4% | 0.0 [-0.1,0.1] | °C/h |
| CRP 24h slope, mean (SD) | 40.2% | 0.0 (2.8) | mg/l/h |
| leukocytes 24h slope, mean (SD) | 20.4% | -0.0 (0.1) | 10^9/l/h |
| thrombocytes 24h slope, mean (SD) | 18.3% | -0.4 (1.9) | 10^9/l/h |
| d-dimer 8h slope, median [Q1,Q3] | 68.7% | 0.0 [0.0,0.0] | ng/ml/h |
| lactate arterial 8h slope, median [Q1,Q3] | 86.4% | 0.0 [-0.0,0.0] | mmol/l/h |
| pO2 arterial 8h slope, median [Q1,Q3] | 81.3% | -0.1 [-1.1,0.9] | mmHg/h |
| pCO2 arterial 8h slope, median [Q1,Q3] | 43.5% | -0.2 [-1.0,0.4] | mmHg/h |
| pH arterial 8h slope, median [Q1,Q3] | 80.1% | 0.0 [-0.0,0.0] | /h |
| base excess 8h slope, median [Q1,Q3] | 76.5% | 0.1 [-0.1,0.2] | mmol/l/h |
| creatinine 24h slope, mean (SD) | 19.5% | -0.2 (2.7) | µmol/l |
| PaO2/FiO2 ratio 3h last, median [Q1,Q3] | 18.3% | 111.6 [86.8,141.7] | |
| ventilatory ratio 3h last, median [Q1,Q3] | 32.2% | 2.2 [1.7,2.9] | |
| mechanical power 3h last, median [Q1,Q3] | 23.5% | 32.0 [20.9,44.7] | J/min |
| lung compliance 3h last, median [Q1,Q3] | 9.8% | 32.8 [24.4,48.5] | ml/cmH2O |
| PaO2/FiO2 ratio difference, median [Q1,Q3] | 27.3% | 14.9 [-5.3,40.5] | |
| ventilatory ratio difference, median [Q1,Q3] | 37.4% | 0.0 [-0.3,0.4] | |
| mechanical power difference, median [Q1,Q3] | 26.7% | 0.2 [-9.8,9.2] | J/min |
| lung compliance difference, median [Q1,Q3] | 12.9% | -0.6 [-5.8,3.6] | ml/cmH2O |
| length of stay, median [Q1,Q3] | 0.0% | 18.4 [12.5,29.6] | days |
| mortality, n (%) | 0.0% | 1714 (47.4) | |

## Supplementary Table 8. ROC AUC score

ROC AUC scores for trained models on various outcome parameters for the relative cut-off of 10% improvement.

|  | **Composite** | **PaO2/FiO2 Ratio** | **Ventilatory Ratio** | **Mechanical Power** | **Compliance** |
| --- | --- | --- | --- | --- | --- |
| LR | 0.563 | 0.619 | 0.592 | 0.663 | 0.597 |
| RF | 0.547 | 0.591 | 0.581 | 0.570 | 0.604 |
| KNN | 0.512 | 0.571 | 0.492 | 0.555 | 0.529 |
| SVM | 0.500 | 0.500 | 0.500 | 0.506 | 0.500 |
| GNB | 0.547 | 0.557 | 0.535 | 0.544 | 0.596 |
| XGB | 0.515 | 0.617 | 0.582 | 0.613 | 0.577 |

## Supplementary Table 9. F1-score

F1 scores for trained models on various outcome parameters for the relative cut-off of 10% improvement.

|  | **Composite** | **PaO2/FiO2 Ratio** | **Ventilatory Ratio** | **Mechanical Power** | **Compliance** |
| --- | --- | --- | --- | --- | --- |
| LR | 0.277 | 0.643 | 0.491 | 0.611 | 0.377 |
| RF | 0.267 | 0.643 | 0.482 | 0.534 | 0.384 |
| KNN | 0.173 | 0.647 | 0.287 | 0.460 | 0.276 |
| SVM | 0.000 | 0.657 | 0.000 | 0.049 | 0.000 |
| GNB | 0.260 | 0.665 | 0.318 | 0.369 | 0.369 |
| XGB | 0.231 | 0.650 | 0.466 | 0.576 | 0.351 |

## Supplementary Table 10. Sensitivity Analysis Minimal Prone Duration

In this sensitivity analysis, no limits were placed on the minimal duration of prone position in contrast to the manuscript analysis which set a default of no response to prone positions with a total duration of less than 4 hours. ROC AUC scores in this sensitivity analysis are comparable to the original analysis.

|  | **Composite** | **PaO2/FiO2 Ratio** | **Ventilatory Ratio** | **Mechanical Power** | **Compliance** |
| --- | --- | --- | --- | --- | --- |
| LR | 0.583 | 0.631 | 0.594 | 0.658 | 0.587 |
| RF | 0.556 | 0.615 | 0.587 | 0.573 | 0.597 |
| KNN | 0.504 | 0.555 | 0.502 | 0.541 | 0.527 |
| SVM | 0.500 | 0.530 | 0.500 | 0.526 | 0.500 |
| GNB | 0.558 | 0.552 | 0.521 | 0.557 | 0.585 |
| XGB | 0.528 | 0.631 | 0.595 | 0.627 | 0.597 |

## Supplementary Table 11. Sensitivity Analysis Cut-off Values

ROC AUC scores for trained models grouped by various cut-off values of relative or absolute improvement.

|  | **Composite** | | **PaO2/FiO2 Ratio** | | **Ventilatory Ratio** | **Mechanical Power** | **Compliance** |
| --- | --- | --- | --- | --- | --- | --- | --- |
|  | **Absolute** | **Relative, 20%** | **Absolute** | **Relative, 20%** | **Relative, 20%** | **Relative, 20%** | **Relative, 20%** |
| LR | 0.601 | 0.556 | 0.683 | 0.665 | 0.574 | 0.688 | 0.674 |
| RF | 0.581 | 0.544 | 0.639 | 0.628 | 0.580 | 0.630 | 0.686 |
| KNN | 0.511 | 0.523 | 0.591 | 0.596 | 0.547 | 0.539 | 0.556 |
| SVM | 0.500 | 0.500 | 0.644 | 0.636 | 0.500 | 0.500 | 0.500 |
| GNB | 0.567 | 0.547 | 0.608 | 0.617 | 0.495 | 0.552 | 0.612 |
| XGB | 0.563 | 0.521 | 0.641 | 0.651 | 0.556 | 0.670 | 0.624 |

## Supplementary Table 12. Ranked Feature Importance

Sorted and ranked feature importance for the most consistently important features from the most predictive models. A higher number reflects a greater relative importance within the model. An average rank across models shows features which consistently contributed to these models.

|  | **LR** | **RF** | **GNB** | **XGB** | **Mean Rank** |
| --- | --- | --- | --- | --- | --- |
| PaO2/FiO2 ratio 3h last | 73 | 73 | 73 | 73 | 73.0 |
| last FiO2 2h slope | 72 | 70 | 64 | 70 | 69.0 |
| PaO2/FiO2 ratio 2h | 65 | 72 | 70 | 44 | 62.8 |
| ventilatory ratio 8h slope | 69 | 49 | 61.5 | 66 | 61.4 |
| pO2 arterial 2h | 61 | 71 | 72 | 36 | 60.0 |
| pCO2 arterial 2h | 37 | 66 | 59 | 68 | 57.5 |
| paCO2-etCO2 2h | 58 | 64 | 67 | 41 | 57.5 |
| O2 saturation 2h | 71 | 67 | 47 | 38 | 55.8 |
| PEEP 2h | 41 | 55 | 61.5 | 50 | 51.9 |
| heart rate 2h | 39 | 57 | 51.5 | 58 | 51.4 |
| ventilatory ratio 3h last | 45 | 46 | 49 | 61 | 50.3 |
| FiO2 2h | 63 | 69 | 16 | 46 | 48.5 |
| lactate arterial 2h | 60 | 29 | 36.5 | 60 | 46.4 |
| creatinine 24h slope | 64 | 65 | 26 | 30 | 46.3 |
| leukocytes 2h | 28 | 58 | 51.5 | 47 | 46.1 |
| pO2 arterial 8h slope | 67 | 13 | 56.5 | 48 | 46.1 |
| respiratory system compliance 3h last | 70 | 35 | 71 | 7 | 45.8 |
| PEEP 8h slope | 62 | 68 | 20.5 | 31 | 45.4 |
| FiO2 8h slope | 52 | 26 | 49 | 54 | 45.3 |
| sodium 2h | 53 | 40 | 65.5 | 21 | 44.9 |
| O2 saturation 8h slope | 59 | 61 | 36.5 | 22 | 44.6 |
| mechanical power 8h slope | 56 | 25 | 38.5 | 57 | 44.1 |
| leukocytes 24h slope | 49 | 37 | 45.5 | 45 | 44.1 |
| pCO2 arterial 8h slope | 50 | 31 | 43.5 | 49 | 43.4 |
| temperature 8h slope | 66 | 22 | 26 | 59 | 43.3 |
| lung compliance 2h | 23 | 36 | 65.5 | 43 | 41.9 |
| minute volume 2h | 30 | 47 | 63 | 23 | 40.8 |
| etCO2 2h | 26 | 62 | 32.5 | 42 | 40.6 |
| respiratory rate 2h | 47 | 34 | 68 | 13 | 40.5 |
| mechanical power 2h | 44 | 38 | 38.5 | 39 | 39.9 |
| ventilatory ratio 2h | 55 | 63 | 13 | 25 | 39.0 |
| peak airway pressure 8h slope | 46 | 43 | 45.5 | 20 | 38.6 |
| creatinine 2h | 68 | 59 | 6 | 18 | 37.8 |
| temperature 2h | 36 | 45 | 54 | 16 | 37.8 |
| responder_failed | 17 | 7 | 60 | 67 | 37.8 |
| mean airway pressure 2h | 2 | 56 | 58 | 32 | 37.0 |
| d-dimer 2h | 38 | 19 | 16 | 71 | 36.0 |
| etCO2 8h slope | 19 | 53 | 6 | 65 | 35.8 |
| minute volume 8h slope | 24 | 41 | 26 | 51 | 35.5 |
| heart rate 8h slope | 57 | 28 | 20.5 | 35 | 35.1 |
| chloride 2h | 51 | 20 | 35 | 34 | 35.0 |
| thrombocytes 2h | 20 | 60 | 29.5 | 29 | 34.6 |
| delta pressure 2h | 54 | 33 | 41 | 9 | 34.3 |
| peak airway pressure 2h | 32 | 48 | 43.5 | 11 | 33.6 |
| mean arterial blood pressure 8h slope | 42 | 24 | 49 | 17 | 33.0 |
| CRP 2h | 1 | 21 | 56.5 | 53 | 32.9 |
| PaO2/FiO2 ratio 8h slope | 14 | 10 | 41 | 62 | 31.8 |
| tidal volume per kg 2h | 6 | 52 | 41 | 27 | 31.5 |
| mean airway pressure 8h slope | 35 | 16 | 20.5 | 52 | 30.9 |
| pH arterial 2h | 43 | 32 | 34 | 14 | 30.8 |
| base excess 8h slope | 34 | 11 | 6 | 72 | 30.8 |
| bmi | 33 | 54 | 6 | 28 | 30.3 |
| respiratory rate 8h slope | 10 | 42 | 6 | 63 | 30.3 |
| pH arterial 8h slope | 22 | 9 | 20.5 | 69 | 30.1 |
| base excess 2h | 18 | 44 | 31 | 26 | 29.8 |
| CRP 24h slope | 48 | 23 | 26 | 19 | 29.0 |
| tidal volume per kg 8h slope | 15 | 30 | 6 | 64 | 28.8 |
| paCO2-etCO2 8h slope | 40 | 12 | 6 | 55 | 28.3 |
| mechanical power 3h last | 29 | 50 | 13 | 10 | 25.5 |
| mean arterial blood pressure 2h | 13 | 51 | 29.5 | 8 | 25.4 |
| age | 27 | 18 | 20.5 | 33 | 24.6 |
| thrombocytes 24h slope | 8 | 39 | 6 | 40 | 23.3 |
| responder_success | 16 | 3 | 16 | 56 | 22.8 |
| nice_other | 25 | 4 | 55 | 3 | 21.8 |
| nice_resp | 9 | 2 | 69 | 5 | 21.3 |
| bilirubin 2h | 5 | 15 | 53 | 6 | 19.8 |
| delta pressure 8h slope | 7 | 17 | 20.5 | 24 | 17.1 |
| albumin 2h | 4 | 14 | 6 | 37 | 15.3 |
| respiratory system compliance 8h slope | 11 | 27 | 6 | 15 | 14.8 |
| gender, female | 31 | 1 | 13 | 12 | 14.3 |
| d-dimer 8h slope | 21 | 8 | 26 | 1 | 14.0 |
| responder_unknown | 3 | 5 | 32.5 | 2 | 10.6 |
| lactate arterial 8h slope | 12 | 6 | 6 | 4 | 7.0 |

## Supplementary Table 13. Feature Importance Values

Feature importances, permutation importances and coefficients for the various models on predicting PaO2/FiO2 ratio. These values describe their contribution to the predictive performance of the models where a negative number directs the model towards failure while a positive number directs towards success.

|  | **LR** | **RF** | **GNB** | **XGB** |
| --- | --- | --- | --- | --- |
| PaO2/FiO2 ratio 3h last | -4.775 | 0.077 | 0.022 | 0.035 |
| last FiO2 2h slope | 2.078 | 0.026 | -0.004 | 0.024 |
| PaO2/FiO2 ratio 2h | -0.926 | 0.047 | 0.008 | 0.014 |
| ventilatory ratio 8h slope | 1.207 | 0.015 | -0.004 | 0.020 |
| pO2 arterial 2h | -0.759 | 0.027 | 0.016 | 0.013 |
| pCO2 arterial 2h | -0.312 | 0.019 | -0.004 | 0.023 |
| paCO2-etCO2 2h | -0.719 | 0.018 | -0.005 | 0.013 |
| O2 saturation 2h | 1.350 | 0.020 | -0.003 | 0.013 |
| PEEP 2h | 0.404 | 0.016 | -0.004 | 0.015 |
| heart rate 2h | -0.363 | 0.016 | -0.003 | 0.015 |
| ventilatory ratio 3h last | 0.429 | 0.014 | -0.003 | 0.017 |
| FiO2 2h | -0.775 | 0.022 | 0.000 | 0.015 |
| lactate arterial 2h | -0.755 | 0.012 | -0.002 | 0.016 |
| creatinine 24h slope | 0.905 | 0.019 | -0.001 | 0.012 |
| leukocytes 2h | -0.267 | 0.017 | -0.003 | 0.015 |
| pO2 arterial 8h slope | -0.990 | 0.007 | -0.004 | 0.015 |
| respiratory system compliance 3h last | -1.268 | 0.012 | -0.009 | 0.008 |
| PEEP 8h slope | -0.772 | 0.021 | -0.001 | 0.012 |
| FiO2 8h slope | -0.575 | 0.011 | -0.003 | 0.015 |
| sodium 2h | -0.586 | 0.014 | -0.005 | 0.011 |
| O2 saturation 8h slope | -0.739 | 0.018 | -0.002 | 0.011 |
| mechanical power 8h slope | -0.644 | 0.011 | -0.002 | 0.015 |
| leukocytes 24h slope | 0.541 | 0.012 | 0.002 | 0.014 |
| pCO2 arterial 8h slope | -0.563 | 0.012 | -0.002 | 0.015 |
| temperature 8h slope | -0.935 | 0.011 | -0.001 | 0.015 |
| lung compliance 2h | 0.212 | 0.012 | -0.005 | 0.014 |
| minute volume 2h | 0.287 | 0.014 | -0.004 | 0.011 |
| etCO2 2h | -0.221 | 0.018 | 0.001 | 0.014 |
| respiratory rate 2h | -0.514 | 0.012 | -0.006 | 0.010 |
| mechanical power 2h | 0.422 | 0.012 | -0.002 | 0.013 |
| ventilatory ratio 2h | -0.637 | 0.018 | 0.000 | 0.012 |
| peak airway pressure 8h slope | -0.485 | 0.014 | 0.002 | 0.011 |
| creatinine 2h | 1.102 | 0.017 | 0.000 | 0.011 |
| temperature 2h | -0.307 | 0.014 | -0.003 | 0.011 |
| responder_failed | -0.145 | 0.003 | 0.004 | 0.021 |
| mean airway pressure 2h | 0.006 | 0.016 | -0.004 | 0.012 |
| d-dimer 2h | -0.344 | 0.010 | 0.000 | 0.028 |
| etCO2 8h slope | -0.184 | 0.016 | 0.000 | 0.019 |
| minute volume 8h slope | 0.216 | 0.014 | -0.001 | 0.015 |
| heart rate 8h slope | -0.717 | 0.012 | -0.001 | 0.013 |
| chloride 2h | 0.567 | 0.010 | -0.001 | 0.013 |
| thrombocytes 2h | 0.189 | 0.017 | 0.001 | 0.012 |
| delta pressure 2h | -0.634 | 0.012 | -0.002 | 0.009 |
| peak airway pressure 2h | -0.294 | 0.014 | -0.002 | 0.009 |
| mean arterial blood pressure 8h slope | -0.406 | 0.011 | -0.003 | 0.011 |
| CRP 2h | -0.003 | 0.011 | -0.004 | 0.015 |
| PaO2/FiO2 ratio 8h slope | -0.117 | 0.005 | -0.002 | 0.017 |
| tidal volume per kg 2h | -0.054 | 0.015 | -0.002 | 0.012 |
| mean airway pressure 8h slope | -0.307 | 0.010 | -0.001 | 0.015 |
| pH arterial 2h | 0.410 | 0.012 | -0.001 | 0.010 |
| base excess 8h slope | -0.306 | 0.006 | 0.000 | 0.032 |
| bmi | -0.297 | 0.016 | 0.000 | 0.012 |
| respiratory rate 8h slope | 0.093 | 0.014 | 0.000 | 0.018 |
| pH arterial 8h slope | -0.197 | 0.004 | -0.001 | 0.024 |
| base excess 2h | -0.159 | 0.014 | 0.001 | 0.012 |
| CRP 24h slope | -0.519 | 0.011 | -0.001 | 0.011 |
| tidal volume per kg 8h slope | 0.125 | 0.012 | 0.000 | 0.018 |
| paCO2-etCO2 8h slope | -0.368 | 0.007 | 0.000 | 0.015 |
| mechanical power 3h last | -0.280 | 0.015 | 0.000 | 0.009 |
| mean arterial blood pressure 2h | -0.104 | 0.015 | 0.001 | 0.009 |
| age | -0.258 | 0.010 | -0.001 | 0.013 |
| thrombocytes 24h slope | -0.063 | 0.013 | 0.000 | 0.013 |
| responder_success | 0.130 | 0.001 | 0.000 | 0.015 |
| nice_other | -0.218 | 0.002 | 0.003 | 0.006 |
| nice_resp | -0.092 | 0.001 | 0.006 | 0.007 |
| bilirubin 2h | 0.039 | 0.008 | 0.003 | 0.007 |
| delta pressure 8h slope | -0.060 | 0.010 | -0.001 | 0.012 |
| albumin 2h | -0.020 | 0.008 | 0.000 | 0.013 |
| respiratory system compliance 8h slope | -0.095 | 0.012 | 0.000 | 0.010 |
| gender, female | 0.294 | 0.001 | 0.000 | 0.009 |
| d-dimer 8h slope | 0.190 | 0.003 | -0.001 | 0.000 |
| responder_unknown | 0.016 | 0.002 | 0.001 | 0.001 |
| lactate arterial 8h slope | 0.100 | 0.002 | 0.000 | 0.007 |

# Figures

## Supplementary Figure 1.

PaO_2_/FiO_2_ ratio difference in mmHg over time after starting prone position. Prone positions which continued for longer than 24 hours were filtered out.
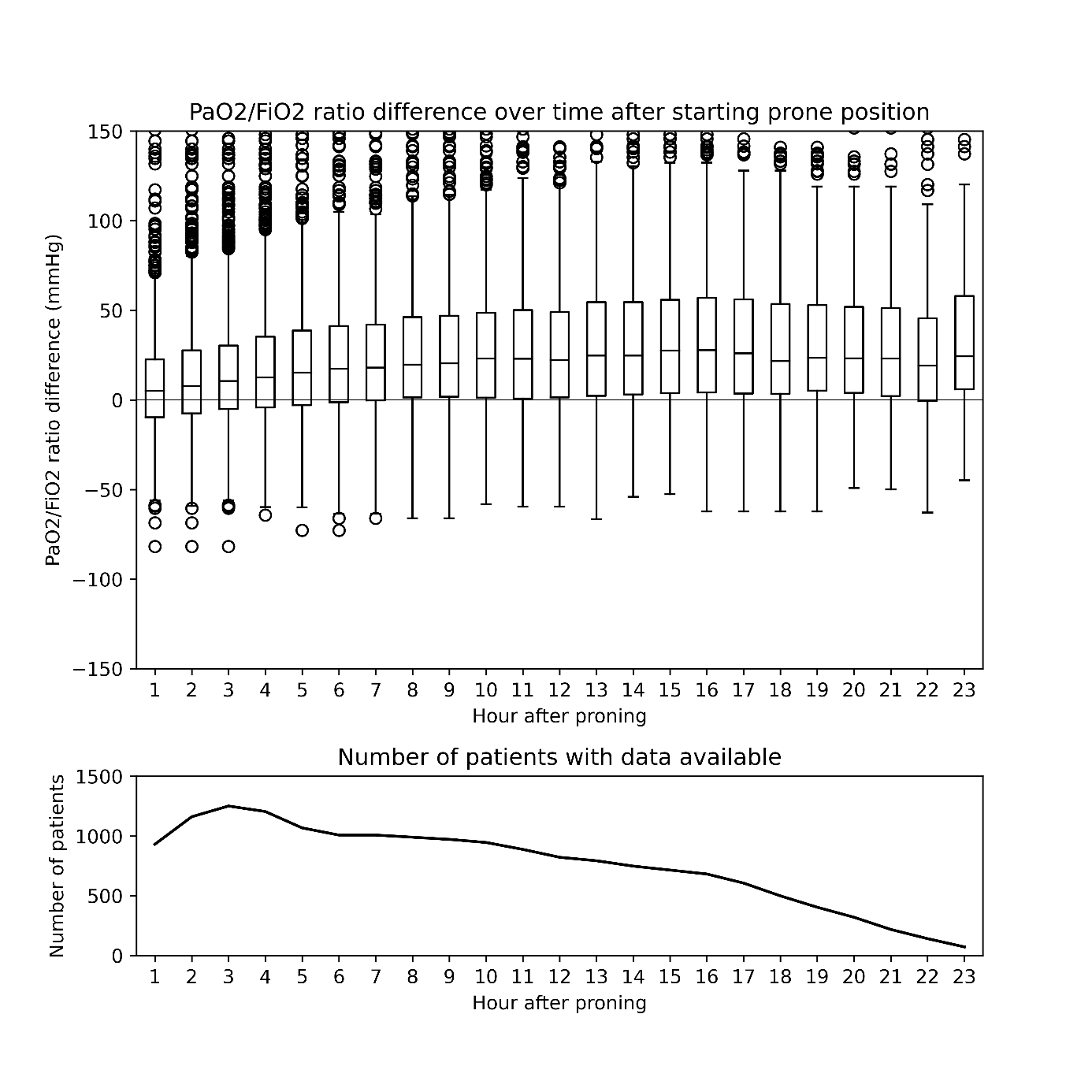


# References

1. Sinha P, Calfee CS, Beitler JR, Soni N, Ho K, Matthay MA, et al. Physiologic Analysis and Clinical Performance of the Ventilatory Ratio in Acute Respiratory Distress Syndrome. Am J Respir Crit Care Med. 2019 Feb 1;199(3):333–41.

2. Giosa L, Busana M, Pasticci I, Bonifazi M, Macrì MM, Romitti F, et al. Mechanical power at a glance: a simple surrogate for volume-controlled ventilation. Intensive Care Med Exp. 2019 Nov 27;7(1):61.

# Acknowledgements

The Dutch ICU Data Sharing Against COVID-19 Collaborators:

From the coordinating group:

Tariq A. Dam, MD, Department of Intensive Care Medicine, Laboratory for Critical Care Computational Intelligence, Amsterdam Medical Data Science, Amsterdam UMC, Vrije Universiteit, Amsterdam, The Netherlands,

Luca F. Roggeveen, MD, Department of Intensive Care Medicine, Laboratory for Critical Care Computational Intelligence, Amsterdam Medical Data Science, Amsterdam UMC, Vrije Universiteit, Amsterdam, The Netherlands,

Fuda van Diggelen, MSc, Quantitative Data Analytics Group, Department of Computer Science, Faculty of Science, VU University, Amsterdam, The Netherlands,

Lucas M. Fleuren, MD, Department of Intensive Care Medicine, Laboratory for Critical Care Computational Intelligence, Amsterdam Medical Data Science, Amsterdam UMC, Vrije Universiteit, Amsterdam, The Netherlands,

Ameet R. Jagesar, MD, Department of Intensive Care Medicine, Laboratory for Critical Care Computational Intelligence, Amsterdam Medical Data Science, Amsterdam UMC, Vrije Universiteit, Amsterdam, The Netherlands,

Martijn Otten, MD, Department of Intensive Care Medicine, Laboratory for Critical Care Computational Intelligence, Amsterdam Medical Data Science, Amsterdam UMC, Vrije Universiteit, Amsterdam, The Netherlands,

Heder J. de Vries, MD, Department of Intensive Care Medicine, Laboratory for Critical Care Computational Intelligence, Amsterdam Medical Data Science, Amsterdam UMC, Vrije Universiteit, Amsterdam, The Netherlands,

Diederik Gommers, MD, PhD, Department of Intensive Care, Erasmus Medical Center, Rotterdam, The Netherlands,

Olaf L. Cremer, MD, PhD, Intensive Care, UMC Utrecht, Utrecht, The Netherlands,

Rob J. Bosman, MD, ICU, OLVG, Amsterdam, The Netherlands,

Sander Rigter, MD, Department of Anesthesiology and Intensive Care, St. Antonius Hospital, Nieuwegein, The Netherlands,

Evert-Jan Wils, MD, PhD, Department of Intensive Care, Franciscus Gasthuis & Vlietland, Rotterdam, The Netherlands,

Tim Frenzel, MD, PhD, Department of Intensive Care Medicine, Radboud University Medical Center, Nijmegen, The Netherlands,

Dave A. Dongelmans, MD, PhD, Department of Intensive Care Medicine, Amsterdam UMC, Amsterdam, The Netherlands,

Remko de Jong, MD, Intensive Care, Bovenij Ziekenhuis, Amsterdam, The Netherlands,

Marco A.A. Peters, MD, Intensive Care, Canisius Wilhelmina Ziekenhuis, Nijmegen, The Netherlands,

Marlijn J.A Kamps, MD, Intensive Care, Catharina Ziekenhuis Eindhoven, Eindhoven, The Netherlands,

Dharmanand Ramnarain, MD, Department of Intensive Care, ETZ Tilburg, Tilburg, The Netherlands,

Ralph Nowitzky, MD, Intensive Care, HagaZiekenhuis, Den Haag, The Netherlands,

Fleur G.C.A. Nooteboom, MD, Intensive Care, Laurentius Ziekenhuis, Roermond, The Netherlands,

Wouter de Ruijter, MD, PhD, Department of Intensive Care Medicine, Northwest Clinics, Alkmaar, The Netherlands,

Louise C. Urlings-Strop, MD, PhD, Intensive Care, Reinier de Graaf Gasthuis, Delft, The Netherlands,

Ellen G.M. Smit, MD, Intensive Care, Spaarne Gasthuis, Haarlem en Hoofddorp, The Netherlands,

D. Jannet Mehagnoul-Schipper, MD, PhD, Intensive Care, VieCuri Medisch Centrum, Venlo, The Netherlands,

Tom Dormans, MD, PhD, Intensive care, Zuyderland MC, Heerlen, The Netherlands,

Cornelis P.C. de Jager, MD, PhD, Department of Intensive Care, Jeroen Bosch Ziekenhuis, Den Bosch, The Netherlands,

Stefaan H.A. Hendriks, MD, Intensive Care, Albert Schweitzerziekenhuis, Dordrecht, The Netherlands,

Sefanja Achterberg, MD, PhD, ICU, Haaglanden Medisch Centrum, Den Haag, The Netherlands,

Evelien Oostdijk, MD, PhD, ICU, Maasstad Ziekenhuis Rotterdam, Rotterdam, The Netherlands,

Auke C. Reidinga, MD, ICU, SEH, BWC, Martiniziekenhuis, Groningen, The Netherlands,

Barbara Festen-Spanjer, MD, Intensive Care, Ziekenhuis Gelderse Vallei, Ede, The Netherlands,

Gert B. Brunnekreef, MD, Department of Intensive Care, Ziekenhuisgroep Twente, Almelo, The Netherlands,

Alexander D. Cornet, MD, PhD, FRCP, Department of Intensive Care, Medisch Spectrum Twente, Enschede, The Netherlands,

Walter van den Tempel, MD, Department of Intensive Care, Ikazia Ziekenhuis Rotterdam, Rotterdam, The Netherlands,

Age D. Boelens, MD, Anesthesiology, Antonius Ziekenhuis Sneek, Sneek, The Netherlands,

Peter Koetsier, MD, Intensive Care, Medisch Centrum Leeuwarden, Leeuwarden, The Netherlands,

Judith Lens, MD, ICU, IJsselland Ziekenhuis, Capelle aan den IJssel, The Netherlands,

Harald J. Faber, MD, ICU, WZA, Assen, The Netherlands,

A. Karakus, MD, Department of Intensive Care, Diakonessenhuis Hospital, Utrecht, The Netherlands,

Robert Entjes, MD, Department of Intensive Care, Adrz, Goes, The Netherlands,

Paul de Jong, MD, Department of Anesthesia and Intensive Care, Slingeland Ziekenhuis, Doetinchem, The Netherlands,

Thijs C.D. Rettig, MD, PhD, Department of Anesthesiology, Intensive Care and Pain Medicine, Amphia Ziekenhuis, Breda, The Netherlands,

Sesmu Arbous, MD, PhD, Intensivist, LUMC, Leiden, The Netherlands,

Sebastiaan J.J. Vonk, MSc, Pacmed, Amsterdam, The Netherlands,

Tomas Machado, Pacmed, Amsterdam, The Netherlands,

Willem E. Herter, BSc, Pacmed, Amsterdam, The Netherlands,

Harm-Jan de Grooth, MD, PhD, Department of Intensive Care Medicine, Laboratory for Critical Care Computational Intelligence, Amsterdam Medical Data Science, Amsterdam UMC, Vrije Universiteit, Amsterdam, The Netherlands,

Patrick J. Thoral, MD, EDIC, Department of Intensive Care Medicine, Laboratory for Critical Care Computational Intelligence, Amsterdam Medical Data Science, Amsterdam UMC, Vrije Universiteit, Amsterdam, The Netherlands,

Armand R.J. Girbes, MD, PhD, EDIC, Department of Intensive Care Medicine, Laboratory for Critical Care Computational Intelligence, Amsterdam Medical Data Science, Amsterdam UMC, Vrije Universiteit, Amsterdam, The Netherlands,

Mark Hoogendoorn, PhD, Quantitative Data Analytics Group, Department of Computer Science, Faculty of Science, VU University, Amsterdam, The Netherlands,

Paul W.G. Elbers, MD, PhD, EDIC, Department of Intensive Care Medicine, Laboratory for Critical Care Computational Intelligence, Amsterdam Medical Data Science, Amsterdam UMC, Vrije Universiteit, Amsterdam, The Netherlands,

From collaborating hospitals having shared data:

Julia Koeter, MD, Intensive Care, Canisius Wilhelmina Ziekenhuis, Nijmegen, The Netherlands,

Roger van Rietschote, Business Intelligence, Haaglanden MC, Den Haag,The Netherlands,

M.C. Reuland, MD, Department of Intensive Care Medicine, Amsterdam UMC, Universiteit van Amsterdam, Amsterdam, The Netherlands,

Laura van Manen, MD, Department of Intensive Care, BovenIJ Ziekenhuis, Amsterdam, The Netherlands,

Leon Montenij, MD, PhD, Department of Anesthesiology, Pain Management and Intensive Care, Catharina Ziekenhuis Eindhoven, Eindhoven, The Netherlands,

Jasper van Bommel, MD, PhD, Department of Intensive Care, Erasmus Medical Center, Rotterdam, The Netherlands,

Roy van den Berg, Department of Intensive Care, ETZ Tilburg, Tilburg, The Netherlands,

Ellen van Geest, Department of ICMT, Haga Ziekenhuis, Den Haag, The Netherlands,

Anisa Hana, MD, PhD, Intensive Care, Laurentius Ziekenhuis, Roermond, The Netherlands,

B. van den Bogaard, MD, PhD, ICU, OLVG, Amsterdam, The Netherlands,

Prof. Peter Pickkers, Department of Intensive Care Medicine, Radboud University Medical Centre, Nijmegen, The Netherlands,

Pim van der Heiden, MD, PhD, Intensive Care, Reinier de Graaf Gasthuis, Delft, The Netherlands,

Claudia (C.W.) van Gemeren, MD, Intensive Care, Spaarne Gasthuis, Haarlem en Hoofddorp, The Netherlands,

Arend Jan Meinders, MD, Department of Internal Medicine and Intensive Care, St Antonius Hospital, Nieuwegein, The Netherlands,

Martha de Bruin, MD, Department of Intensive Care, Franciscus Gasthuis & Vlietland, Rotterdam, The Netherlands,

Emma Rademaker, MD, MSc, Department of Intensive Care, UMC Utrecht, Utrecht, The Netherlands,

Frits H.M. van Osch, PhD, Department of Clinical Epidemiology, VieCuri Medisch Centrum, Venlo, The Netherlands,

Martijn de Kruif, MD, PhD, Department of Pulmonology, Zuyderland MC, Heerlen, The Netherlands,

Nicolas Schroten, MD, Intensive Care, Albert Schweitzerziekenhuis, Dordrecht, The Netherlands,

Klaas Sierk Arnold, MD, Anesthesiology, Antonius Ziekenhuis Sneek, Sneek, The Netherlands,

J.W. Fijen, MD, PhD, Department of Intensive Care, Diakonessenhuis Hospital, Utrecht, The Netherland,

Jacomar J.M. van Koesveld, MD, ICU, IJsselland Ziekenhuis, Capelle aan den IJssel, The Netherlands,

Koen S. Simons, MD, PhD, Department of Intensive Care, Jeroen Bosch Ziekenhuis, Den Bosch, The Netherlands,

Joost Labout, MD, PhD, ICU, Maasstad Ziekenhuis Rotterdam, The Netherlands,

Bart van de Gaauw, MD, Martini ziekenhuis, Groningen, The Netherlands,

Michael Kuiper, Intensive Care, Medisch Centrum Leeuwarden, Leeuwarden, The Netherlands,

Albertus Beishuizen, MD, PhD, Department of Intensive Care, Medisch Spectrum Twente, Enschede, The Netherlands,

Dennis Geutjes, Department of Information Technology, Slingeland Ziekenhuis, Doetinchem, The Netherlands,

Johan Lutisan, MD, ICU, WZA, Assen, The Netherlands,

Bart P. Grady, MD, PhD, Department of Intensive Care, Ziekenhuisgroep Twente, Almelo, The Netherlands,

Remko van den Akker, Intensive Care, Adrz, Goes, The Netherlands,

Tom A. Rijpstra, MD, Department of Anesthesiology, Intensive Care and Pain Medicine, Amphia Ziekenhuis, Breda, The Netherlands,

Wim G. Boersma, MD, PhD, Department of Pulmonology, Northwest Clinics, Alkmaar, The Netherlands,

From collaborating hospitals having signed the data sharing agreement:

Daniël Pretorius, MD, Department of Intensive Care Medicine, Hospital St Jansdal, Harderwijk, The Netherlands,

Menno Beukema, MD, Department of Intensive Care, Streekziekenhuis Koningin Beatrix, Winterswijk, The Netherlands,

Bram Simons, MD, Intensive Care, Bravis Ziekenhuis, Bergen op Zoom en Roosendaal, The Netherlands,

A.A. Rijkeboer, MD, ICU, Flevoziekenhuis, Almere, The Netherlands,

Marcel Aries, MD, PhD, MUMC+, University Maastricht, Maastricht, The Netherlands,

Niels C. Gritters van den Oever, MD, Intensive Care, Treant Zorggroep, Emmen, The Netherlands,

Martijn van Tellingen, MD, EDIC, Department of Intensive Care Medicine, afdeling Intensive Care, ziekenhuis Tjongerschans, Heerenveen, The Netherlands,

Annemieke Dijkstra, MD, Department of Intensive Care Medicine, Het Van Weel-Bethesda Ziekenhuis, Dirksland, The Netherlands,

Rutger van Raalte, Department of Intensive Care, Tergooi hospital, Hilversum, The Netherlands,

From the Laboratory for Critical Care Computational Intelligence:

Ali el Hassouni, PhD, Quantitative Data Analytics Group, Department of Computer Science, Faculty of Science, VU University, Amsterdam, The Netherlands,

David Romero Guzman, PhD, Quantitative Data Analytics Group, Department of Computer Science, Faculty of Science, VU University, Amsterdam, The Netherlands,

Sandjai Bhulai, PhD, Analytics and Optimization Group, Department of Mathematics, Faculty of Science, Vrije Universiteit, Amsterdam, The Netherlands,

Dagmar M. Ouweneel, PhD, Department of Intensive Care Medicine, Laboratory for Critical Care Computational Intelligence, Amsterdam Medical Data Science, Amsterdam UMC, Vrije Universiteit, Amsterdam, The Netherlands,

Ronald Driessen, Department of Intensive Care Medicine, Laboratory for Critical Care Computational Intelligence, Amsterdam Medical Data Science, Amsterdam UMC, Vrije Universiteit, Amsterdam, The Netherlands,

Jan Peppink, Department of Intensive Care Medicine, Laboratory for Critical Care Computational Intelligence, Amsterdam Medical Data Science, Amsterdam UMC, Vrije Universiteit, Amsterdam, The Netherlands,

G.J. Zijlstra, MD, PhD, Department of Intensive Care Medicine, Laboratory for Critical Care Computational Intelligence, Amsterdam Medical Data Science, Amsterdam UMC, Vrije Universiteit, Amsterdam, The Netherlands,

A.J. van Tienhoven, MD, Department of Intensive Care Medicine, Laboratory for Critical Care Computational Intelligence, Amsterdam Medical Data Science, Amsterdam UMC, Vrije Universiteit, Amsterdam, The Netherlands,

Evelien van der Heiden, MD, Department of Intensive Care Medicine, Amsterdam Medical Data Science, Amsterdam UMC, Vrije Universiteit, Amsterdam, The Netherlands,

Jan Jaap Spijkstra, MD, PhD, Department of Intensive Care Medicine, Amsterdam Medical Data Science, Amsterdam UMC, Vrije Universiteit, Amsterdam, The Netherlands,

Hans van der Spoel, MD, Department of Intensive Care Medicine, Amsterdam Medical Data Science, Amsterdam UMC, Vrije Universiteit, Amsterdam, The Netherlands,

Angelique M.E. de Man, MD, PhD, Department of Intensive Care Medicine, Amsterdam Medical Data Science, Amsterdam UMC, Vrije Universiteit, Amsterdam, The Netherlands,

Thomas Klausch, PhD, Department of Clinical Epidemiology, Laboratory for Critical Care Computational Intelligence, Amsterdam Medical Data Science, Amsterdam UMC, Vrije Universiteit, Amsterdam, The Netherlands,

From Pacmed:

Robbert C.A. Lalisang, MD, Pacmed, Amsterdam, The Netherlands,

Michele Tonutti, MRes, Pacmed, Amsterdam, The Netherlands,

Daan P. de Bruin, MSc, Pacmed, Amsterdam, The Netherlands,

Mattia Fornasa, PhD, Pacmed, Amsterdam, The Netherlands,

Michael de Neree tot Babberich, Pacmed, Amsterdam, The Netherlands,

Olivier Thijssens, MSc, Pacmed, Amsterdam, The Netherlands,

Lot Wagemakers, Pacmed, Amsterdam, The Netherlands,

Hilde G.A. van der Pol, Pacmed, Amsterdam, The Netherlands,

Tom Hendriks, Pacmed, Amsterdam, The Netherlands,

Julie Berend, Pacmed, Amsterdam, The Netherlands,

Virginia Ceni Silva, Pacmed, Amsterdam, The Netherlands,

Robert F.J. Kullberg, MD, Pacmed, Amsterdam, The Netherlands,

Taco Houwert, MSc, Pacmed, Amsterdam, The Netherlands,

Hidde Hovenkamp, MSc, Pacmed, Amsterdam, The Netherlands,

Roberto Noorduijn Londono, MSc, Pacmed, Amsterdam, The Netherlands,

Davide Quintarelli, MSc, Pacmed, Amsterdam, The Netherlands,

Martijn G. Scholtemeijer, MD, Pacmed, Amsterdam, The Netherlands,

Aletta A. de Beer, MSc, Pacmed, Amsterdam, The Netherlands,

Giovanni Cinà, PhD, Pacmed, Amsterdam, The Netherlands,

Adam Izdebski, Pacmed, Amsterdam, The Netherlands,

From RCCnet:

Leo Heunks, MD, PhD, Department of Intensive Care Medicine, Amsterdam Medical Data Science, Amsterdam UMC, Vrije Universiteit, Amsterdam, The Netherlands,

Nicole Juffermans, MD, PhD, ICU, OLVG, Amsterdam, The Netherlands,

Arjen J.C. Slooter, MD, PhD, Department of Intensive Care Medicine, UMC Utrecht, Utrecht University, Utrecht, the Netherlands,

From other collaborating partners:

Martijn Beudel, MD, PhD, Department of Neurology, Amsterdam UMC, Universiteit van Amsterdam, Amsterdam, The Netherlands,
